# Effects of Using High‐Frequency TENS During an Exercise Therapy Program in Knee Osteoarthritis: A Randomized Sham‐Controlled Trial

**DOI:** 10.1002/pri.70351

**Published:** 2026-09-23

**Authors:** Patrícia Gabrielle dos Santos, Richard Eloin Liebano, Bruno Ruocco Verengue, Aron Charles Barbosa da Silva, Barbara Greco Miura, Carlos Eduardo Girasol, Almir Vieira Dibai‐Filho, Cid André Fidelis‐de‐Paula‐Gomes

**Affiliations:** ^1^ Postgraduate Program in Rehabilitation Sciences Universidade Nove de Julho São Paulo Brazil; ^2^ Department of Rehabilitation Sciences University of Hartford West Hartford Connecticut USA; ^3^ Department of Physiotherapy Universidade do Estado de Santa Catarina Florianópolis Brazil; ^4^ Postgraduate Program in Physical Education Universidade Federal do Maranhão São Luís Maranhão Brazil

**Keywords:** exercise therapy, knee osteoarthritis, pain, transcutaneous electrical nerve stimulation

## Abstract

**Background and Purpose:**

Therapeutic exercise is central to knee osteoarthritis (KOA) management, but movement‐evoked pain may limit adherence and outcomes. High‐frequency transcutaneous electrical nerve stimulation may reduce pain during movement and enhance exercise‐related outcomes. This study evaluated whether high‐frequency transcutaneous electrical nerve stimulation applied during an exercise therapy program provides additional benefits over sham stimulation in individuals with KOA.

**Methods:**

In this randomized, sham‐controlled superiority trial with intended participant blinding and blinded outcome assessment, 96 individuals with KOA were randomized to exercise therapy plus active high‐frequency transcutaneous electrical nerve stimulation or exercise therapy plus sham transcutaneous electrical nerve stimulation. The primary outcome was the Knee Injury and Osteoarthritis Outcome Score Pain subscale. Secondary outcomes included the remaining Knee Injury and Osteoarthritis Outcome Score subscales, pain intensity, pain‐related self‐efficacy, patient‐specific function, disability, quadriceps isometric strength, and physical function. Outcomes were assessed at baseline, post‐intervention, and 1 month follow‐up. Linear mixed models were used for analysis.

**Results:**

Active high‐frequency transcutaneous electrical nerve stimulation did not provide additional benefit over sham stimulation for the Knee Injury and Osteoarthritis Outcome Score Pain subscale at post‐intervention (adjusted mean difference, 1.05 points; 95% CI, −5.19 to 7.29) or at 1 month follow‐up (0.40 points; 95% CI, −5.91 to 6.71). These confidence intervals did not reach the predefined 10‐point minimal clinically important difference. No statistically significant between‐group differences were observed for any secondary outcome. No adverse effects were reported.

**Discussion:**

Adding high‐frequency transcutaneous electrical nerve stimulation to a structured exercise therapy program did not confer additional benefits over sham stimulation in individuals with KOA. These findings should be interpreted in light of the absence of formal blinding assessment, the short follow‐up, and the absence of an exercise‐only group.

**Trial Registration:**

This trial was registered at ClinicalTrials.gov on December 14, 2023, under the identifier NCT06184451

## 1 Introduction

Osteoarthritis is a common chronic disease of synovial joints, characterized by dysregulated cellular metabolism and extracellular matrix breakdown that trigger maladaptive repair responses (Collins et al. 2026). Knee osteoarthritis (KOA) affects about 30% of adults aged 45 years and older and is a major cause of persistent pain and disability. These symptoms may be further exacerbated by pain‐related fear, leading to avoidance, physical deconditioning, and distress (Katz et al. 2021; Kise et al. 2025).

Exercise is a core treatment for KOA, aiming to improve lower‐limb muscle mass, neuromotor control, and joint range of motion, thereby increasing strength, reducing pain, and improving physical function (Yan et al. 2025). However, exercise‐ and movement‐evoked pain may limit adherence and compromise outcomes, making pain‐relieving strategies relevant before or during exercise. In this context, transcutaneous electrical nerve stimulation (TENS) is a promising adjunct, as systematic reviews suggest, for reducing pain and knee‐related dysfunction and may improve outcomes when combined with other interventions (Wu et al. 2022; Johnson et al. 2022). Because its analgesic effect is greatest during and immediately after stimulation, high‐frequency TENS (HF‐TENS) may be particularly suitable during exercise, given its rapid onset of analgesia and potential to improve muscle activation, force generation, and central excitability during movement (Sluka et al. 2013; Son et al. 2017).

We hypothesized that HF‐TENS applied during the exercise therapy program (ETP) would yield greater improvements in knee pain‐related symptoms, pain intensity, pain‐related self‐efficacy, patient‐specific function, disability, quadriceps isometric strength, and physical function than sham TENS. Therefore, this study aimed to assess the effects of HF‐TENS during an ETP, compared with sham TENS, on knee pain‐related symptoms, pain intensity, pain‐related self‐efficacy, patient‐specific function, disability, quadriceps isometric strength, and physical function in individuals with KOA.

## 2 Methods

### 2.1 Study Design

This superiority randomized clinical trial with two parallel arms, intended participant blinding, and blinded outcome assessment was conducted from February 2024 to August 2025 at the laboratory of Nove de Julho University, São Paulo, Brazil. The Human Research Ethics Committee of Nove de Julho University, São Paulo, Brazil, approved the study on November 22, 2023 (Protocol No. 75610223.5.0000.5511), and the trial was registered at ClinicalTrials.gov on December 14, 2023 (identifier NCT06184451).

All participants received oral and written study information and provided verbal and written informed consent. The study adhered to the Declaration of Helsinki, the CONSORT guidelines, the OARSI recommendations for rehabilitation trials in knee osteoarthritis, and the TIDieR guidelines (Hoffmann et al. 2014; Fitzgerald et al. 2015; Hopewell et al. 2025).

Participants were recruited from the university rehabilitation clinic and two primary healthcare units, as well as through referrals from physiotherapists in São Paulo, Brazil.

### 2.2 Participants

Participants of both sexes, aged 40 to 75 years, were eligible if they had unilateral symptomatic knee osteoarthritis, symptoms lasting at least 3 months, knee pain > 3 on the Numeric Pain Rating Scale (NPRS), morning stiffness < 30 min, crepitus, bony tenderness without palpable warmth, and radiographic KOA classified as Kellgren and Lawrence grades 1–3 (Alfredo et al. 2024).

Exclusion criteria included hip symptoms as the primary source of pain, any condition that could confound KOA symptoms, severe osteoporosis, fibromyalgia, oncologic history, active inflammatory joint disease, prior lower‐limb joint replacement, neurological disorders, infected wounds or knee osteomyelitis, deep vein thrombosis or thrombophlebitis, lower‐limb sensory impairment, cognitive impairment, cardiopulmonary limitations to exercise, use of a walking aid, recent knee trauma, physiotherapy within the previous 6 months, intra‐articular corticosteroid injections, anti‐inflammatory drugs, opioid medications, or chondroprotective agents (Fitzgerald et al. 2015).

### 2.3 Randomization and Concealed Allocation

The clinical research coordinator, who was solely responsible for this phase of the study, generated the random allocation sequence in Microsoft Excel (Microsoft Corporation, Washington) using 24 blocks of four codes to ensure a 1:1 allocation ratio between groups. Allocation concealment was maintained using opaque, sealed, sequentially numbered envelopes numbered 1–96. The same coordinator monitored adherence, exercise progression, protocol deviations, potential adverse events, and concomitant care weekly, using individualized calendars, attendance logs, and phone or text‐message contact.

Four investigators were involved in the trial procedures. Two conducted the assessments, and two administered the interventions, one per study arm. All had more than 15 years of experience in chronic musculoskeletal pain management and completed study‐specific training 3 months before data collection to standardize procedures and ensure adherence to the protocol.

Because of the nature of the interventions, treating physiotherapists could not be blinded. Outcome assessors were blinded to group assignment. Participants were informed that they would receive one of two types of electrical stimulation during the ETP, but they were not told whether the stimulation was active or sham. No formal assessment of blinding success was conducted.

All sessions were held in a private room at the university laboratory, with 30 min intervals between appointments to minimize contact among participants. Participants were also asked not to disclose details of the intervention or study procedures.

### 2.4 Outcome Measures

The assessments were conducted at three time points: baseline, post‐intervention (5 weeks), and 1 month follow‐up (1‐moFU), except for physical function and quadriceps isometric strength, which were not assessed at follow‐up. All participants completed a researcher‐developed questionnaire to collect demographic and clinical data.

The primary outcome was knee pain‐related symptoms, assessed using the Knee Injury and Osteoarthritis Outcome Score (KOOS) Pain subscale (Almeida et al. 2022). The KOOS is a 42‐item patient‐reported measure with 5 subscales, scored from 0 to 100, with higher scores indicating better knee‐related status. The Brazilian Portuguese version has demonstrated adequate validity, reliability, and responsiveness in individuals with knee osteoarthritis, and the recommended MCID for KOOS Pain is 10 points (Mills et al. 2016).

Secondary outcomes included the remaining KOOS subscales (Symptoms, Activities of Daily Living, Sport and Recreation Function, and knee‐related Quality of Life) (Almeida et al. 2022), as well as pain intensity, pain‐related self‐efficacy, patient‐specific function, disability, quadriceps isometric strength, and physical function. The recommended MCID values for the KOOS secondary subscales are six points for Symptoms, six for Activities of Daily Living, eight for Sport and Recreation Function, and 10 for knee‐related Quality of Life (Mills et al. 2016).

Pain intensity was assessed using the NPRS, which ranged from 0 (no pain) to 10 (worst pain imaginable). Participants rated pain over the previous 7 days, both at rest and after movement. An MCID of two points was used (Farrar et al. 2001).

Pain self‐efficacy was assessed using the Pain Self‐Efficacy Questionnaire (PSEQ), a 10‐item scale rated 0–6, yielding a total score of 0–60; higher scores indicate stronger self‐efficacy beliefs. An MCID of 11.5 points was used (Dube et al. 2021).

Patient‐specific function was assessed using the Patient‐Specific Functional Scale (PSFS), in which participants identified three priority activities and rated their ability to perform each on a 1–10 scale relative to the pre‐intervention level. An MCID of 1.5 points for the mean of the three activities was used (Moore et al. 2020).

Disability was assessed using the 12‐item World Health Organization Disability Assessment Schedule 2.0 (WHODAS 2.0), which evaluates disability on a 1–5 Likert scale, with total scores ranging from 12 to 60, where higher scores indicate greater disability. An MCID of nine points was used (Katajapuu et al. 2020).

Quadriceps isometric strength was measured as the maximal voluntary isometric contraction using a portable dynamometer (Lafayette Manual Muscle System, Model 01163). Participants were seated with the hip flexed at 90° and the knee at 60°. The dynamometer was secured anteriorly at the malleolar level around the distal tibia with a nonelastic strap during knee extension. Strength values were normalized to body weight using the formula kg muscle strength/kg body mass ×  100 (de Paula Gomes et al. 2018). An MCID threshold of 6% was applied (Ruhdorfer et al. 2015).

Physical function was assessed using the 30s sit‐to‐stand (30s‐STS) test, which recorded the number of repetitions completed in 30 s, with standardized instructions and a standardized chair. Participants started seated, with feet flat on the floor and arms crossed over the chest, and were instructed to stand to full hip and knee extension, then return. An MCID of 2.5 repetitions was adopted (Mostafaee et al. 2024).

### 2.5 Interventions

Interventions took place over 5 weeks. Sessions were held twice a week on nonconsecutive days, with each session lasting about 90 min. All equipment used in the study was calibrated and met established technical standards.

The ETP + active HF‐TENS group received an exercise therapy program comprising warm‐up, resistance, neuromuscular, mobility, and balance exercises (Table S1), based on a previous study and on functional movements commonly reported as difficult by individuals with KOA (Anderson et al. 2019; Benner et al. 2019; Hislop et al. 2020). The exercise menu was standardized, but progression was individualized. Participants were reassessed every 2 weeks. When exercises were completed without symptom exacerbation, loads were increased to 40%–60% of 1‐repetition maximum, and elastic‐band resistance was selected to allow 12 controlled repetitions without worsening pain (Anderson et al. 2019). Exercise dose, attendance, reasons for missed sessions, progression decisions, and safety issues were recorded at each session. Perceived exertion was monitored clinically through therapist observation and participant feedback, but a formal rating of perceived exertion scale was not used.

During each ETP session, active HF‐TENS was delivered via a portable Neurodyn TENS device (Ibramed) as part of the exercise therapy program (Table S2) (Vance et al. 2012; Inal et al. 2016; B. Pietrosimone et al. 2020; Shi et al. 2021).

Before group‐specific procedures, participants were briefly familiarized with the stimulation process and the study‐specific visual analog tool developed by the authors, the TENS Sensory Perception Scale. Intensity was checked every 5 min, or sooner, to adjust the pulse amplitude. The 1–10 scale, colored from blue to red, indicated sensory perception and the ability to perform the prescribed exercises during stimulation. Participants reported only the number and color, and the investigator confirmed tolerability and task performance using standardized questions. The target level was 6–8, representing the maximum tolerated intensity that could elicit a visible muscle contraction without pain or discomfort.

For the ETP + sham TENS group, participants completed the same ETP. Sham stimulation was delivered using the same device and electrode placement as in the active HF‐TENS group, but the current was automatically disabled after 30 s.

Participants in both groups were asked not to start additional physiotherapy, intra‐articular injections, or other knee‐related interventions during the study period and to avoid changes to analgesic or anti‐inflammatory medications whenever possible. Any medication use, new treatment, missed session, or adverse event was reviewed at treatment visits and during weekly phone or text‐message contact and recorded as a potential protocol deviation.

### 2.6 Statistical Analysis

The sample size was determined for the primary outcome, KOOS Pain. A between‐group difference of about 10 points was considered clinically significant based on available estimates of clinically important change/difference for KOOS in individuals with KOA undergoing non‐surgical treatment (Mills et al. 2016). Assuming a standard deviation of 15 points, 80% power, and a two‐sided alpha of 0.05, 37 participants per group were needed to detect this difference (Mills et al. 2016). To account for an expected 20% attrition rate, the sample size was increased to 48 participants per group. The calculation was performed using Ene software, version 3.0 (Universitat Autònoma de Barcelona, Spain).

An intention‐to‐treat analysis was conducted at the 5% significance level for all comparisons. Data normality was assessed using histograms, which indicated that all variables were normally distributed. Descriptive statistics are reported as means and SDs. Adjusted mean differences (MDs) and 95% confidence intervals (CIs) were estimated using linear mixed models with fixed effects for group, time, and the group‐by‐time interaction. These models used all available repeated outcome data and accommodated missing post‐intervention or follow‐up observations under the missing‐at‐random assumption, without last‐observation‐carried‐forward or multiple imputation. Thus, all randomized participants contributed to the analyses based on the data available for each outcome. No sensitivity analyses were performed. Between‐group differences reported in the tables are model‐based, adjusted estimates and may not equal simple arithmetic differences between unadjusted within‐group change scores. Statistical analyses were performed using SPSS version 23.0 (IBM Corp., Armonk, NY, USA).

## 3 Results

### 3.1 General Characteristics of the Subjects

**FIGURE 1:**
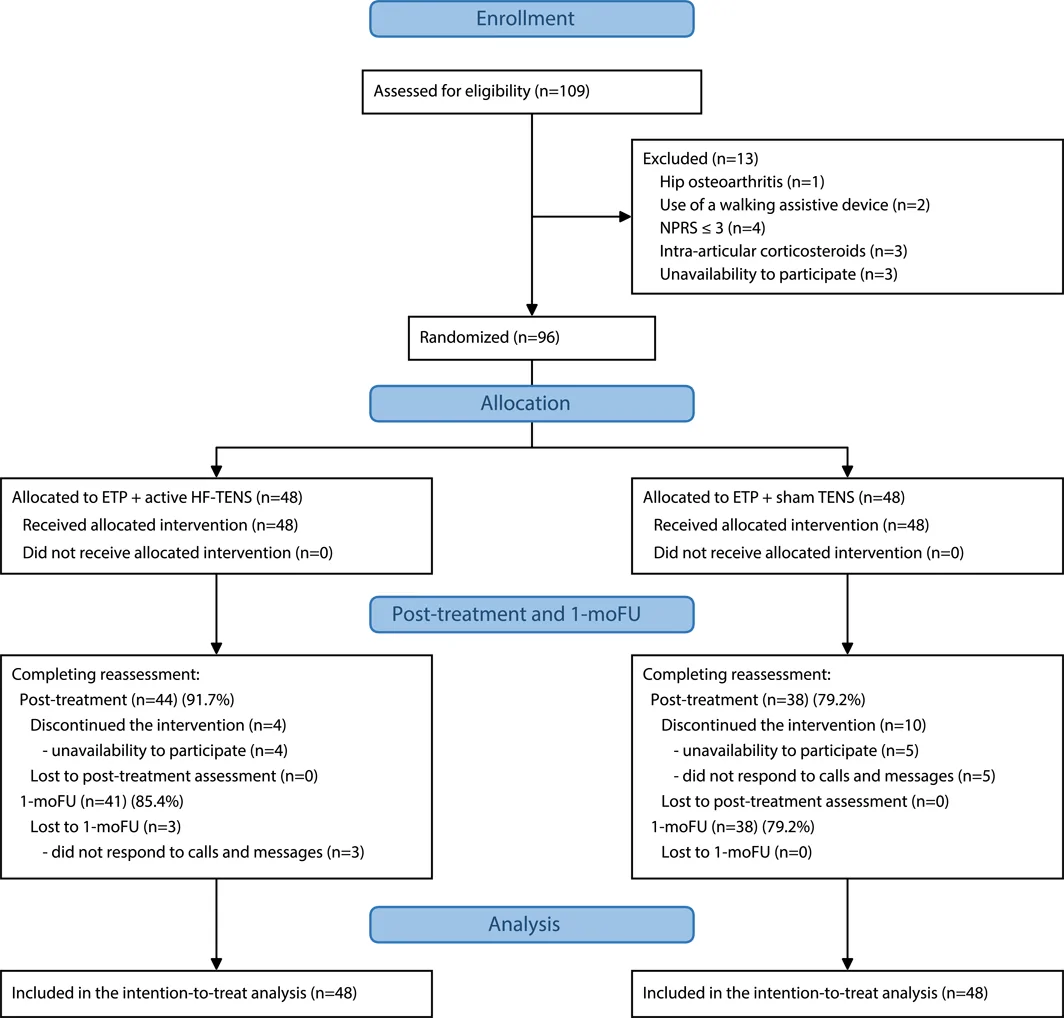
Flow chart of participants through the trial. 1‐moFU, 1‐month follow‐up; ETP + active HF‐TENS, exercise therapy program plus high‐frequency active transcutaneous nerve stimulation; ETP + sham TENS, exercise therapy program plus sham transcutaneous electrical nerve stimulation; NPRS, Numeric Pain Rating Scale.

Of 109 screened participants, 96 were randomized and received the allocated intervention, with 48 in each group. Post‐intervention and 1 month follow‐up assessments were completed by 44 and 41 participants in the active HF‐TENS group, and by 38 participants at both time points in the sham TENS group. Baseline characteristics were comparable between groups (Figure 1 and Table 1). Four participants (8.3%) in the active HF‐TENS group and 10 (20.8%) in the sham TENS group discontinued the intervention and attended no further sessions, so the 10 planned sessions were completed by 44 participants (91.7%) and 38 participants (79.2%), respectively. Because the participants who discontinued the intervention were the same participants who did not attend the post‐intervention assessment, these adherence proportions coincide with the proportions completing that assessment. No crossovers, major protocol deviations, additional knee physiotherapy, intra‐articular injections, changes in analgesic or anti‐inflammatory medication, or adverse effects were reported (Table 2).

**TABLE 1:** Baseline characteristics of the participants.

Variable	ETP + active HF‐TENS (*n* = 48)	ETP + sham TENS (*n* = 48)
Age (y), mean (SD)	62 (9.84)	59 (11.36)
Women, *n* (%)	44 (91.67)	45 (93.75)
Weight (kg), mean (SD)	75.15 (10.31)	77.4 (14.31)
Height (cm), mean (SD)	1.65 (0.09)	1.66 (0.09)
BMI (kg/m^2^), mean (SD)	27.71 (4.52)	28.20 (5.13)
Marital status, *n* (%)		
Single	9 (18.8)	19 (39.6)
Married	19 (39.6)	20 (41.7)
Divorced	10 (20.8)	5 (10.4)
Widowed	10 (20.8)	4 (8.3)
Education, *n* (%)		
Elementary school complete	3 (6.3)	6 (12.5)
High school complete	25 (52.1)	24 (50)
University complete	13 (27.1)	15 (31.3)
University incomplete	3 (6.3)	1 (2.1)
Not declared	4 (8.3)	2 (4.2)
Diagnosis time (years), mean (SD)	8 (6.44)	6 (3.86)
Affected knee, *n* (%)		
Right	34 (70.8)	33 (68.8)
Left	14 (29.2)	15 (31.3)

Abbreviations: BMI, body mass index; ETP + active HF‐TENS, exercise therapy program plus high‐frequency active transcutaneous electrical nerve stimulation; ETP + sham TENS, exercise therapy program plus sham transcutaneous electrical nerve stimulation; kg, kilogram; m, meters; SD, standard deviation.

**TABLE 2:** Intervention adherence, fidelity, concomitant care, and safety.

Variable	ETP + active HF‐TENS (*n* = 48)	ETP + sham TENS (*n* = 48)
Planned intervention sessions	10 per participant	10 per participant
Participants receiving allocated intervention	48/48 (100%)	48/48 (100%)
Participants completing the 10 planned intervention sessions, *n*/*N* (%)	44/48 (91.7%)	38/48 (79.2%)
Discontinued the intervention, *n*/*N* (%)	4/48 (8.3%)	10/48 (20.8%)
Completed post‐intervention assessment	44/48 (91.7%)	38/48 (79.2%)
Completed 1‐month follow‐up assessment	41/48 (85.4%)	38/48 (79.2%)
Crossovers or major protocol deviations	0 reported	0 reported
Additional knee physiotherapy or intra‐articular injections during the study	0 reported	0 reported
Adverse effects	0 reported	0 reported

*Note:* The participants who discontinued the intervention were the same participants who did not attend the post‐intervention assessment, and no participant who completed the 10 planned sessions missed that assessment. The proportions reported for completion of the intervention and for completion of the post‐intervention assessment therefore coincide.

Abbreviations: ETP, exercise therapy program; HF‐TENS, high‐frequency transcutaneous electrical nerve stimulation.

### 3.2 Within‐Group Comparisons

Both groups showed within‐group improvements over time in several patient‐reported outcomes. Changes in the KOOS subscales exceeded the corresponding MCID at both time points in both groups, except for KOOS Pain at the 1 month follow‐up, where the changes of 9.28 and 9.58 points fell just below the 10‐point threshold (Table 3). Improvements in patient‐specific function, pain‐related self‐efficacy, disability, and physical function were not clinically meaningful. Only the ETP + sham TENS group showed a statistically significant within‐group increase in physical function from baseline to post‐intervention.

**TABLE 3:** Mean (SD) of groups, mean (SD) within‐group differences, and adjusted between‐group mean differences (95% CI) estimated from the linear mixed models.

Outcomes	Groups						Within‐group differences (unadjusted change scores)				Between‐group differences (adjusted mean difference, 95% CI, from linear mixed model)		MCID
	Baseline		Post		1‐moFU		Post minus baseline		1‐moFU minus baseline		Post minus baseline	1‐moFU minus baseline	
	ETP + active HF‐TENS (*n* = 48)	ETP + sham TENS (*n* = 48)	ETP + active HF‐TENS (*n* = 44)	ETP + sham TENS (*n* = 38)	ETP + active HF‐TENS (*n* = 41)	ETP + sham TENS (*n* = 38)	ETP + active HF‐TENS	ETP + sham TENS	ETP + active HF‐TENS	ETP + sham TENS	ETP + active HF‐TENS vs. ETP + sham TENS	ETP + active HF‐TENS vs. ETP + sham TENS	
Knee pain‐related symptoms													
Pain	51.10 (14.70)	47.86 (15.03)	64.02 (13.22)	60.01 (20.09)	63.14 (15.52)	59.21 (19.47)	11.62* (14.92)	10.38* (18.06)	9.28* (14.35)	9.58^*^ (16.89)	1.05 (−5.19 to 7.29)	0.40 (−5.91 to 6.71)	10
Symptoms	59.45 (15.75)	50.45 (17.33)	68.91 (11.46)	59.77 (19.04)	68.55 (11.52)	61.09 (18.98)	9.17* (13.50)	7.24* (15.09)	7.84* (13.05)	8.55* (13.48)	1.37 (−4.11 to 6.86)	−0.44 (−5.99 to 5.10)	6
Activities of daily living	51.81 (14.94)	51.13 (14.98)	61.76 (13.14)	61.42 (18.10)	58.93 (13.01)	63.12 (17.13)	10.16* (14.26)	7.28* (17.97)	6.31* (14.76)	8.98* (17.90)	1.42 (−4.87 to 7.71)	−3.22 (−9.59 to 3.15)	6
Sport and recreation	8.65 (12.75)	9.58 (13.08)	18.52 (20.07)	22.24 (23.01)	18.17 (19.03)	22.37 (26.22)	10.23* (16.70)	10.79* (21.73)	9.88* (14.85)	10.92* (24.27)	−1.54 (−9.21 to 6.13)	−1.94 (−9.70 to 5.82)	8
Quality of life	27.08 (13.36)	30.73 (15.89)	42.61 (16.85)	44.57 (21.21)	42.23 (17.88)	45.72 (21.11)	15.34* (16.86)	11.51* (20.53)	14.02* (17.99)	12.66* (21.43)	2.83 (−4.44 to 10.10)	1.36 (−5.99 to 8.72)	10
Pain intensity last 7 days	5.6 (2.03)	6.29 (1.80)	3.91 (1.96)	4.11 (2.47)	4.10 (2.27)	4.03 (2.42)	−1.5 (2.36)	−1.76 (2.26)	−1.1 (2.23)	−1.84 (2.48)	0.39 (−0.59 to 1.38)	0.73 (−0.26 to 1.73)	2
Pain intensity at rest	4.13 (2.51)	4.90 (2.54)	3.09 (2.11)	3.34 (2.58)	3.24 (2.02)	3.34 (2.56)	−0.89 (1.85)	−1.29 (2.49)	−0.56 (2.50)	−1.29 (2.24)	0.45 (−0.48 to 1.38)	0.67 (−0.28 to 1.62)	
Pain intensity after movement	6.44 (2.08)	6.33 (2.07)	4.43 (2.27)	4.29 (2.60)	4.63 (2.11)	4.5 (2.15)	−1.86 (2.54)	−1.60 (2.47)	−1.49 (2.50)	−1.39 (2.30)	−0.09 (−1.10 to 0.92)	−0.08 (−1.10 to 0.94)	
Patient‐specific function	3.72 (1.21)	3.24 (1.26)	5.09 (1.52)	4.74 (1.98)	4.85 (1.60)	4.83 (2.10)	1.28* (1.63)	1.27* (2.03)	0.98* (1.74)	1.37* (2.37)	−0.07 (−0.81 to 0.67)	−0.37 (−1.12 to 0.38)	1.5
Pain self‐efficacy	43.54 (8.81)	37.90 (10.01)	49.27 (6.35)	43.47 (11.22)	47.59 (6.60)	44.37 (9.63)	5.34* (7.66)	4.58* (9.96)	3.22* (8.57)	5.47* (9.19)	0.51 (−3.03 to 4.05)	−2.07 (−5.65 to 1.51)	11.5
Disability	27.08 (7.10)	30.58 (8.51)	23.43 (5.05)	24.63 (6.69)	25.02 (7.04)	24.84 (6.49)	−3.32* (4.99)	−4.32* (7.93)	−1.90* (5.96)	−4.10* (7.95)	1.60 (−1.32 to 4.51)	2.84 (−0.11 to 5.80)	9
Quadriceps isometric strength	9.89 (2.72)	10.34 (3.39)	10.57 (2.14)	10.79 (2.66)	—	—	0.5 (2.60)	0.55 (1.97)	—	—	0.04 (−0.99 to 1.08)	—	6%
Physical function	9.99 (3.44)	9.15 (2.11)	10.73 (2.75)	10.08 (1.88)	—	—	0.48 (1.99)	0.75* (1.60)	—	—	−1.12 (−2.64 to 0.40)	—	2.5

*Note:* All between‐group differences reported in this table are adjusted mean differences (95% CI) estimated from the linear mixed models, with fixed effects for group, time, and the group‐by‐time interaction. They are model‐based estimates and may not equal the simple arithmetic differences between the unadjusted within‐group change scores presented in the preceding columns. Within‐group change scores were computed in the participants with data available at the corresponding time point, whereas the group means include all randomized participants at baseline, so the change scores may not equal the difference between the group means reported in the preceding columns.

Abbreviations: 1‐moFU, 1‐month follow‐up; Disability‐WHODAS, World Health Organization Disability Assessment Schedule (Range of 12–60 points, higher scores = higher level of disability); ETP + active HF‐TENS, exercise therapy program plus high‐frequency active transcutaneous electrical nerve stimulation; ETP + sham TENS, exercise therapy program plus sham transcutaneous electrical nerve stimulation; Knee pain‐related symptoms‐KOOS, Knee Injury and Osteoarthritis Outcome Score (Range of 0–100 points, high score = fewer symptoms); MCID, Minimal Clinically Important Difference; Pain intensity‐NPRS, Numeric Pain Rating Scale (Range of 0–10 points, high score = higher level of pain intensity); Patient‐specific function‐PSFS, Patient Specific Functional Scale (Range of 1–10 points, higher score = better function); Pain self‐efficacy‐PSEQ, pain self‐efficacy questionnaire (Range of 0–60 points, high score = greater pain self‐efficacy); Physical function‐30‐s sit‐to‐stand; Quadriceps isometric strength‐Maximal voluntary isometric contraction.

^*^ *p* < 0.05.

### 3.3 Between‐Group Comparisons

**FIGURE 2:**
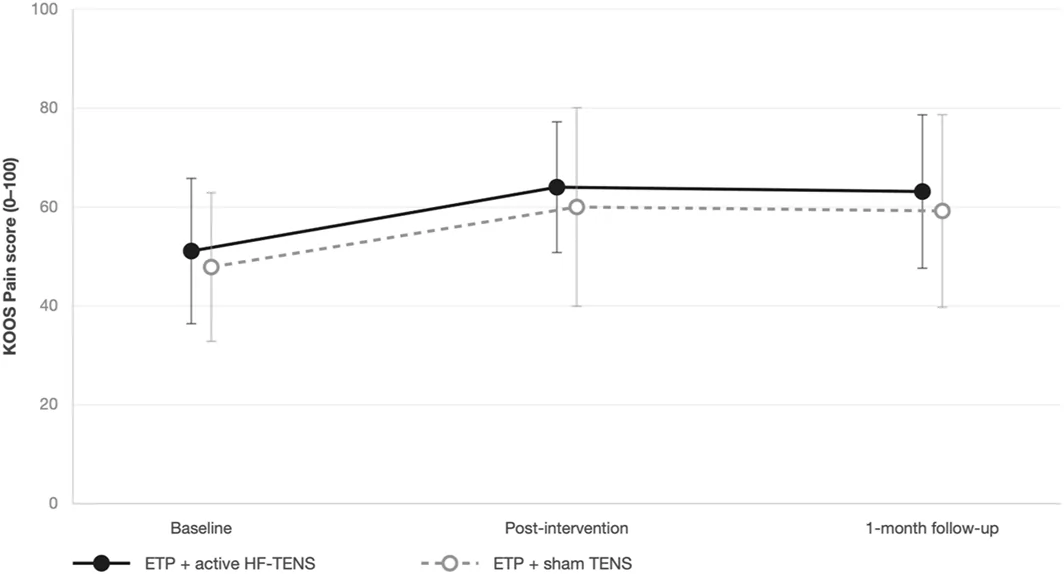
Primary outcome: Knee Injury and Osteoarthritis Outcome Score Pain subscale at baseline, post‐intervention (5 weeks), and 1 month follow‐up in the ETP + active HF‐TENS and ETP + sham TENS groups. Higher scores indicate less pain (range 0–100). Points are group means; error bars represent ± 1 SD and are horizontally offset to reduce overlap. Both groups improved significantly from baseline (*p* < 0.05), with no statistically significant between‐group difference at any time point. 1‐mo FU, 1‐month follow‐up; CI, confidence interval; ETP, exercise therapy program; HF‐TENS, high‐frequency transcutaneous electrical nerve stimulation; KOOS, Knee Injury and Osteoarthritis Outcome Score; TENS, transcutaneous electrical nerve stimulation; SD, standard deviation.

**FIGURE 3:**
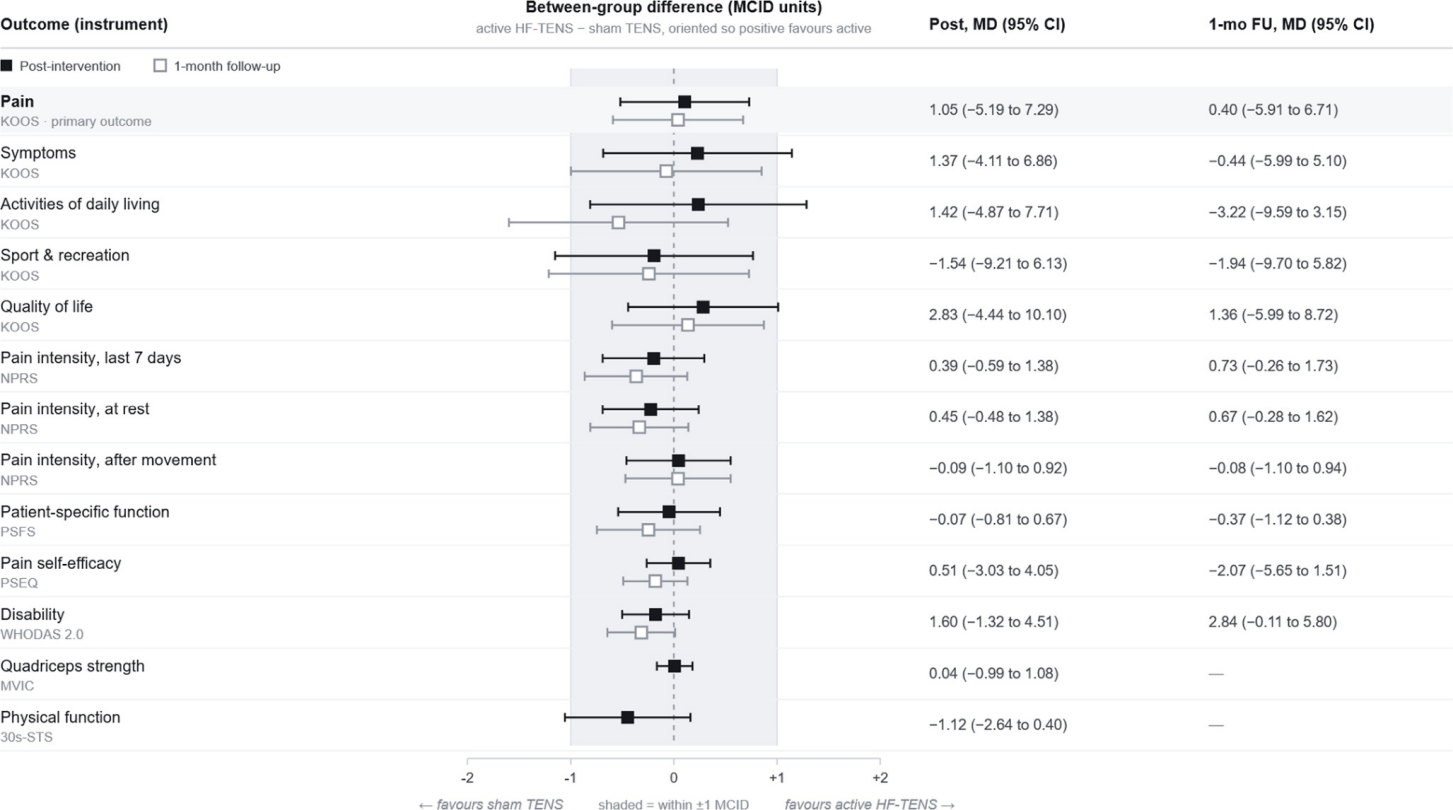
Adjusted between‐group differences (active HF‐TENS minus sham TENS) at post‐intervention and 1‐month follow‐up for every outcome, estimated from linear mixed models. To enable comparison across instruments with different scales, effects are expressed in units of each outcome's minimal clinically important difference (MCID) and oriented so that positive values favor active HF‐TENS. Squares are point estimates and horizontal lines are 95% CIs; the shaded band spans ± 1 MCID (the zone of no clinically important difference) and the dashed line marks no difference (0). Raw mean differences (95% CI) in original units are reported at right. No between‐group difference reached statistical or clinical significance. Quadriceps strength and physical function were not assessed at follow‐up. 30s‐STS, 30‐second sit‐to‐stand; MVIC, maximal voluntary isometric contraction; NPRS, Numeric Pain Rating Scale; PSEQ, Pain Self‐Efficacy Questionnaire; PSFS, Patient‐Specific Functional Scale; WHODAS 2.0, World Health Organization Disability Assessment Schedule.

No statistically significant between‐group differences were observed for the primary outcome, KOOS Pain, at post‐intervention (adjusted mean difference 1.05 points, 95% CI −5.19 to 7.29) or at 1 month follow‐up (0.40 points, 95% CI −5.91 to 6.71). These CIs were below the predefined 10‐point MCID for KOOS Pain, supporting the absence of a clinically important between‐group benefit for the primary outcome. No statistically significant between‐group differences were observed for any secondary outcome at the available time points, including the remaining KOOS subscales, pain intensity, pain‐related self‐efficacy, patient‐specific function, disability, quadriceps isometric strength, and physical function. The trajectory of KOOS Pain scores over time is shown in Figures 2 and 3 and in Table 3.

## 4 Discussion

The main finding of this trial was that HF‐TENS applied during an ETP did not confer additional benefit over sham TENS for knee pain‐related symptoms in individuals with knee osteoarthritis, and no between‐group differences were observed for secondary outcomes. For the primary outcome, the adjusted between‐group estimates were small and the 95% CIs did not include the predefined 10‐point MCID at post‐intervention or 1 month follow‐up. This supports the interpretation that adding active HF‐TENS did not produce a clinically important advantage over sham TENS for KOOS Pain. This study adds to the literature by evaluating HF‐TENS delivered during a structured ETP under sham‐controlled conditions in a clinically relevant sample of knee osteoarthritis.

Interpretation of secondary outcomes should be more cautious. Point estimates were consistently small and did not show a coherent advantage for active HF‐TENS. However, some CIs were wider than those for the primary outcome, and for isolated KOOS subscales, they approached or crossed predefined MCID thresholds. Therefore, the data do not support a clear additional clinical benefit of active HF‐TENS, although clinically meaningful effects for every secondary endpoint cannot be completely excluded.

A systematic review (De Espíndula Brehm et al. 2024) highlighted that evidence supporting the use of TENS during functional activities is limited by small sample sizes and emphasized the need for well‐designed studies comparing active and sham stimulation. Although smaller trials included in that review (Lawson et al. 2022) reported immediate pain reduction during specific functional tasks in individuals with knee osteoarthritis, our trial provides a larger and more comprehensive evaluation over an extended follow‐up period. However, our results showed no additional symptomatic or functional benefit when active HF‐TENS was combined with an ETP (Lawson et al. 2022; De Espíndula Brehm et al. 2024).

This pattern aligns with prior research. In a gait training protocol, adding TENS did not confer additional benefits, whereas hybrid electrical stimulation showed more favorable effects on pressure pain thresholds (Matsuse et al. 2022). Similarly, most studies investigating TENS use during therapeutic exercise found no superiority over placebo for function, disability, muscle strength, or pain intensity among individuals with KOA or older adults with chronic pain (B. G. Pietrosimone et al. 2010, 2011; B. Pietrosimone et al. 2020; Vaillancourt et al. 2021). One study reported greater quadriceps voluntary activation with TENS during exercise but no between‐group differences in WOMAC outcomes (B. G. Pietrosimone et al. 2011). Likewise, a 10‐session trial found no overall benefit from adding TENS to structured exercise, except for short‐term improvements in pain and stiffness (B. Pietrosimone et al. 2020).

This lack of superiority also extends to biomechanical outcomes. Increases in gait speed during TENS application were not sustained at follow‐up (B. G. Pietrosimone et al. 2010), and the immediate hypoalgesia observed in older adults with chronic pain did not translate into long‐term improvements in pain or depression (Vaillancourt et al. 2021). Methodologically, these TENS trials are similar to our study, particularly in their use of continuous, high‐frequency, biphasic stimulation at strong but tolerable intensities, although treatment doses and patient groups varied (B. G. Pietrosimone et al. 2010, 2011; B. Pietrosimone et al. 2020; Vaillancourt et al. 2021).

A plausible explanation is the strong influence of contextual factors in nonpharmacological treatments for knee osteoarthritis, in which much of the observed pain relief may reflect placebo effects, natural history, and cointerventions rather than the specific physiological action of the modality (Chen et al. 2020). This may help explain why adding TENS to a structured ETP rarely provides clear clinical advantages over sham, suggesting that TENS is better viewed as a short‐term aid for movement tolerance and exercise adherence rather than as a strategy expected to confer substantial benefit beyond a well‐structured ETP (Chen et al. 2020).

Another possible explanation is that the TENS protocol remained largely unchanged over time, whereas the ETP was progressively adjusted based on participants' performance. Repeated exposure to the same TENS dose may lead to analgesic tolerance, and previous studies suggest that alternating frequencies or gradually increasing intensity may delay the onset of this tolerance (DeSantana et al. 2008; Liebano et al. 2011).

Although this evidence comes mainly from experimental settings or contexts outside knee osteoarthritis, it suggests that a more adaptable dosing strategy may better align with exercise progression and preserve analgesic responsiveness over time. Because this trial assessed post‐intervention and short‐term follow‐up outcomes rather than responses during or immediately after each session, our findings do not exclude short‐term analgesic benefits during exercise, when TENS is likely to be most effective (Johnson et al. 2022). Movement‐evoked pain is a distinct and meaningful clinical outcome that end‐of‐treatment assessments may not capture, so HF‐TENS may still have made some sessions more tolerable without producing measurable between‐group differences in later outcomes (Son et al. 2017; Fullwood et al. 2021).

This study has limitations that must be acknowledged. No formal assessment of blinding fidelity was performed, and participant blinding may have been imperfect because sham stimulation was discontinued after 30 s, whereas active HF‐TENS continued throughout the exercise session. This issue is particularly relevant because several outcomes were patient‐reported and could be influenced by expectations or perceived treatment credibility. However, any expectancy effect would plausibly favor active HF‐TENS, which was not observed. The 1 month follow‐up period limited our understanding of long‐term effects. Including a control group that performed only ETP could have enhanced internal validity and more clearly identified the specific effects of TENS. In addition, exercise intensity was progressed using repetition targets, 1RM ranges, pain response, and therapist judgment, but formal rating of perceived exertion data were not collected. Recruiting participants from healthcare facilities in a single region may limit applicability to other populations and settings.

Future research should investigate the immediate and intra‐session effects of TENS during ETP, determine optimal stimulation parameters and dosing strategies for individuals with KOA, include formal blinding assessment and session‐by‐session perceived exertion monitoring, and incorporate patient‐centered measures, such as treatment preferences and perceived pain relief during movement.

In conclusion, our findings indicated that adding HF‐TENS to a structured ETP did not confer additional benefits over sham TENS for knee osteoarthritis‐related symptoms, pain intensity, pain‐related self‐efficacy, patient‐specific function, disability, quadriceps isometric strength, and physical function.

## 5 Implications of Physiotherapy Practice

Structured ETP should remain a core treatment for knee osteoarthritis. In this trial, adding HF‐TENS to an ETP provided no additional benefit over sham TENS for pain‐related symptoms, pain intensity, pain‐related self‐efficacy, patient‐specific function, disability, quadriceps isometric strength, and physical function. Physiotherapists should therefore prioritize well‐structured ETPs and consider TENS mainly as a possible short‐term adjunct to improve movement tolerance, rather than as a strategy expected to improve overall clinical outcomes.

## Author Contributions

All authors have made substantial contributions to all three of the sections below: Conception and design of the study or acquisition of data, or analysis and interpretation of data, drafting the article or revising it critically for important intellectual content, and final approval of the version to be submitted. Specifics: This study was designed by P.G.d.S., A.V.D.‐F., and C.A.F.‐d.‐P.‐G. The experiments were performed by P.G.d.S., A.C.B.d.S., B.R.V., and B.G.M. Data validation and resource management were carried out by P.G.d.S. and A.C.B.d.S. Data were analyzed by C.A.F.‐d.‐P.‐G. and P.G.d.S., and the results were critically examined by all authors. The original draft was primarily written by C.A.F.‐d.‐P.‐G. and P.G.d.S., and the manuscript was reviewed and edited by A.V.D.‐F., R.E.L., and C.E.G. C.A.F.‐d.‐P.‐G. was responsible for project supervision.

## Funding

This study was supported by Coordenação de Aperfeiçoamento de Pessoal de Nível Superior, Brasil (CAPES), Finance Code 001. The authors certify that the grant sponsor was not involved in study design, data collection, analysis, and interpretation of data; in the writing of the manuscript; or in the decision to submit the manuscript for publication.

## Ethics Statement

Ethical approval was obtained from the Human Research Ethics Committee of Nove de Julho University in São Paulo, Brazil (Protocol No. 75610223.5.0000.5511), approved on November 22, 2023.

## Consent

Informed consent was obtained from all participants prior to any study procedures.

## Conflicts of Interest

The authors declare no conflicts of interest.

## Permission to Reproduce Material From Other Source

The authors have nothing to report.

## Supporting information


**Table S1:** Exercise therapeutic program description.

**Table S2:** Characteristics of the HF active TENS and sham TENS interventions.

## Data Availability

Data supporting the findings of this study are available in the Open Science Framework repository (available at https://archive.org/details/osf‐registrations‐c34er‐v1 or https://osf.io/pnkms). Additional study materials are available from the corresponding author upon reasonable request.
