# Supplementary material for: Effects of Using High‐Frequency TENS During an Exercise Therapy Program in Knee Osteoarthritis: A Randomized Sham‐Controlled Trial
Source: Physiother Res Int. 2026 Sep 23;31(4):e70351. doi: 10.1002/pri.70351 (PMC13598731; doi:10.1002/pri.70351)
Supplement: Supplementary file 1 [file PRI-31-e70351-s001.docx]

Table S2. Characteristics of the HF active TENS and sham TENS interventions.

| Parameter | HF active TENS | sham TENS |
| --- | --- | --- |
| Device | Portable Neurodyn TENS device, Ibramed^®^ | Portable Neurodyn TENS device, Ibramed^®^ |
| Waveform | Symmetrical biphasic waveform | Symmetrical biphasic waveform |
| Frequency | 150 Hz | 150 Hz |
| Pulse duration | 150 µs | 150 µs |
| Intensity | Maximum tolerated intensity sufficient to elicit a visible muscle contraction, without pain or discomfort | Perceptible stimulation for 30 s, followed by automatic discontinuation of current |
| Mode | High-frequency TENS | Sham TENS |
| Electrode size | 5 × 5 cm | 5 × 5 cm |
| Electrode type/fixation | Self-adhesive electrode | Self-adhesive electrode |
| Number of electrodes | 4 | 4 |
| Electrode placement | Around the painful knee | Around the painful knee |
| Session duration | Applied during the ETP | Applied during the ETP |
| Total exposure time | Approximately 90 minutes per session | Approximately 90 minutes per session |
| Time between amplitude adjustments | Every 5 minutes or whenever requested | Not applicable |

HF: High-frequency, ETP: exercise therapy program, Hz: hertz; µs: microseconds; cm: centimeters.
