# Supplementary material for: Effects of Using High‐Frequency TENS During an Exercise Therapy Program in Knee Osteoarthritis: A Randomized Sham‐Controlled Trial
Source: Physiother Res Int. 2026 Sep 23;31(4):e70351. doi: 10.1002/pri.70351 (PMC13598731; doi:10.1002/pri.70351)
Supplement: Supplementary file 2 [file PRI-31-e70351-s002.docx]

Table S1. Exercise therapeutic program description.

| **Exercise** | **Exercise description** | **How to perform** |
| --- | --- | --- |
| Warm-up | Warm-up on a treadmill (Inbramed®, Master TOP 18), stationary bike (Matrix^®^, upright), or marching in place for 10 minutes. Performed at low intensity. | Treadmill at zero incline, with a standardized speed between 2.0 and 4.0 km/h or cycling on a stationary bike (50 to 70 rpm; resistance levels 1 to 5). |
| Horizontal leg press | The subject was seated with the lower back in contact with the seat, feet positioned on the platform shoulder-width apart, knees flexed at 90°, and hands on the equipment’s upper handles (Technogym^®^, Selection). The seat angle was set at 45°. Following instructions, participants performed a purely concentric action and returned slowly to the starting position before the next repetition. A 1-second pause occurred between the eccentric and concentric phases. An experienced researcher assisted in maintaining the participant's feet on the platform during both phases and provided verbal encouragement throughout the movements. | Three sets of 8-12 repetitions each, with 90-second rest intervals. |
| Leg extension | The subject was seated on the leg extension machine (Nakagym^®^) with the lower back in contact with the seat and ankles positioned against the machine's pad. The height was adjusted according to limb length. The range of motion started at 90° and ended at 45° of knee flexion. A researcher provided verbal encouragement during the movement and assisted in maintaining the participant's proper alignment and joint angles. | Three sets of 8-12 repetitions each, with 90-second rest intervals. |
| Lateral squat walk | Participants stood upright and performed a squat to 45° of knee flexion followed by hip abduction while moving laterally, with an elastic resistance band positioned above the knees. A researcher assisted in maintaining the participant's proper alignment and joint angles. | Three sets of 8-12 repetitions each, with 90-second rest intervals. |
| Step up | The subject standing in an upright position, placed one foot on a step (MBFit^®^) followed by the other to perform the step-up. Hands were placed either on a wall bar or on the hips. The exercise was performed unilaterally, starting with the affected side. The researcher provided verbal commands to instruct and guide the participant through the movement. | Three sets of 8-12 repetitions each, with 90-second rest intervals. |
| Lateral step down | The subject standing in an upright position on a step (MBFit^®^), performed a lateral step-down without touching the foot to the floor while maintaining the other foot supported on the step. Hands were placed either on wall bars or on the hips. Sets were performed bilaterally in a non-alternating manner. The researcher provided verbal commands to instruct and guide the participant through the movement. | Three sets of 8-12 repetitions each, with 90-second rest intervals. |
| Sit to stand | Participants stood upright and performed hip and knee flexion to sit on a chair. Arms were preferably crossed over the chest. The researcher provided verbal commands for the controlled performance of sitting and standing without hand support, whenever possible. | Three sets of 8-12 repetitions each, with 90-second rest intervals. |
| Cool-down | Participants stood upright and performed active stretching of the knee extensors and flexors, using a wall bars chair for support if necessary. | 30-60 seconds for each limb and/or muscle group. |

Progression and fidelity monitoring**:** Exercise selection was standardized, but load, resistance, range, and support were individualized according to symptoms and performance. Participants were reassessed every 2 weeks. Loads progressed to 40% to 60% of one-repetition maximum when exercises were completed without worsening pain. Elastic-band resistance was selected to allow 12 controlled repetitions without symptom exacerbation. Attendance, missed sessions, reasons for absence, progression decisions, safety issues, and protocol deviations were recorded at each session. Formal rating of perceived exertion was not recorded.
